# A 10-month cluster-randomized trial to shift towards plant-forward meals in early childhood education and care centres: effects on bone and mineral metabolism in Finnish children

**DOI:** 10.1007/s00394-026-04063-y

**Published:** 2026-07-23

**Authors:** Suvi T. Itkonen, Jelena Meinilä, Sari Niinistö, Susanna Raulio, Henna Vepsäläinen, Liisa Korkalo, Satu Kinnunen, Mari Åkerlund, Tuuli E. Korhonen, Terhi Vihervaara, Leena Forma, Merja Saarinen, Suvi M. Virtanen, Maijaliisa Erkkola

**Affiliations:** 1 Department of Food and Nutrition, University of Helsinki, Helsinki, Finland; 2 Department of Public Health, Finnish Institute for Health and Welfare, Helsinki, Finland; 3 Faculty of Social Sciences, Tampere University, Tampere, Finland; 4 Institute for Nutrition and Health Research, Helsinki, Finland; 5 Department of Government Services, Finnish Institute for Health and Welfare, Helsinki, Finland; 6 Department of Health and Social Management, University of Eastern Finland, Kuopio, Finland; 7 Natural Resources Institute Finland, Helsinki, Finland; 8 Centre for Child Health Research and Wellbeing Services County of Pirkanmaa, Tampere University Hospital, Tampere, Finland; 9 Tampere Center for Child, Adolescent and Maternal Health Research (TamCAM), Tampere University and Tampere University Hospital, Tampere, Finland

**Keywords:** Daycare, Bone turnover, Vitamin D, Plant-based, Calcium, Protein

## Abstract

**Purpose:**

A shift towards predominantly plant-based diets is emphasized for health and environmental sustainability, but it may affect nutrient adequacy and bone health. We investigated the effects of plant-forward meals in Finnish early childhood education and care (ECEC) centres on bone metabolism and intakes of bone nutrients in 3–5-year-old children.

**Methods:**

ECEC centres were cluster-randomized for 10 months into 11 intervention (more plant-based products and less red meat served and moderation in milk consumption encouraged; n = 55 children for individual-level study) and 12 control (maintained regular meals; n = 61 children) ECEC centres. In the intervention arm, 18 children and in the control arm 28 children provided complete (baseline and endpoint) blood samples and dietary data (food frequency questionnaire). Circulating biomarkers (e.g. serum tartrate-resistant acid phosphatase 5b [S-TRAP5b], total and bone-specific alkaline phosphatase [S-ALP; S-BAP], 25-hydroxyvitamin D [S-25(OH)D], and plasma parathyroid hormone) and intakes of calcium, vitamin D, phosphorus, and protein were analysed using mixed models.

**Results:**

The intervention group had higher bone formation marker S-BAP (0.05 log-U/L, 95% CI 0.004–0.104; *p* = 0.04), bone resorption marker S-TRAP5b (2.12 U/L, 95% CI 0.35–3.90; *p* = 0.02), and S-ALP (0.034 log-U/L, 95% CI 0.002–0.066; *p* = 0.04) concentrations than the control group. No effects on other biomarkers or any nutrient intakes were observed. Mean S-25(OH)D concentrations were adequate (≥ 50 nmol/L).

**Conclusions:**

The clinical relevance of the results indicating accelerated bone turnover without observing any effects on the intakes of critical nutrients requires further investigations to avoid potential harmful consequences for long-term bone health with the shift to plant-forward diets.

**Trial registration:**

ClinicalTrials.gov, NCT05249946. Registered 29 November 2021, https://clinicaltrials.gov/ct2/show/NCT05249946.

**Supplementary Information:**

The online version contains supplementary material available at https://doi.org/10.1007/s00394-026-04063-y.

## Background

Switching towards more plant-based diets is advantageous; in addition to their nutritional benefits, followed by a lower risk of cardiovascular disease and cancers [1, 2], the environmental impacts of the diet decrease as well [3, 4]. However, this dietary change may decrease the intakes of nutrients critical for bone health, such as vitamin D and calcium, since plants are not their primary sources [5–7]. As a consequence, low vitamin D status can lead to lower calcium absorption and subsequently to bone loss due to secondary hyperparathyroidism, i.e. increased parathyroid hormone (PTH) concentrations [8].

Studies on vegan and vegetarian diets in adults have systematically shown lower bone mineral density and higher fracture risk [9–12] as well as higher bone resorption marker and catabolic PTH concentrations [13–15] relative to omnivores. The effects of partial substitution of animal-sourced proteins with plant-sourced ones on bone turnover among Finnish adults have been evaluated in two trials [16, 17]. The first trial focused on partial replacement of animal-based protein sources (including vitamin D-fortified dairy products) with 50–70% total protein originating from different non-fortified plant-based foods for 12 weeks and showed lower calcium and vitamin D intakes and accelerated bone turnover, which can be harmful in the long run [16]. In the second trial, 20% of total protein intake from red and processed meat was replaced with non-soy legume products, while 5% of total protein intake still originated from red and processed meat; vitamin D and calcium intakes did not change and no effects on bone turnover were observed [17]. Thus, the nutritional adequacy of plant-forward dietary transitions depends critically on what foods are replaced and what substitutes are chosen.

Children following plant-based diets have been investigated less, but, for instance, 7–10-year-old Polish children following vegan or vegetarian diets have shown lower bone mineral content or density [18, 19] as well as higher concentrations of bone resorption markers [18, 20, 21] than children following omnivorous diet. Importantly, a recent study observed a linear trend towards higher PTH concentrations in more plant-based diets among 2–7-year-old Finnish children following vegan, vegetarian, and omnivorous diets [22]. The phenomenon was present despite similar and adequate vitamin D status and calcium intakes in the groups, and no differences in bone turnover marker concentrations were detected. This may raise a further concern of the dietary transition from the bone health point of view since high PTH concentrations are catabolic for bone [8].

The recently updated Nordic and Finnish nutrition recommendations stress the importance of a decrease in red and processed meat and moderation in dairy consumption, while increasing the use of local fish and legumes [4, 23]. The consumption of legumes and fish among Finnish preschoolers is limited, whereas milk and red meat are highly consumed [24], indicating that the current dietary habits do not align well with the recommendations. Meanwhile, studies have shown that the early childhood education and care (ECEC) environment is an important determinant of childhood health behaviour [25, 26]. Thus, dietary shifts in ECEC are required to gain health benefits and to increase the environmental sustainability of the diets among the future generations. Simultaneously, it is crucial to evaluate the effects of decreased consumption of key animal-sourced foods on the intakes of potentially compromised nutrients. Moreover, it is necessary to focus on potential metabolic changes that can affect long-term outcomes, such as bone health, the foundation of which is established already in childhood.

Thus, we aimed to investigate how transitioning to plant-forward meals in Finnish ECEC centres affects bone and mineral metabolism and intakes of bone-related nutrients in 3–5-year-old children during a 10-month cluster-randomized intervention.

## Methods

### Study design and randomization

FoodStep, a system-level intervention study at ECEC centres, was conducted in one municipality located in Päijät-Häme (Southern Finland) and three municipalities located in South Ostrobothnia (Western Finland) [27]. Of the 23 participating ECEC centres, recruited in 2021, 15 were located in Päijät-Häme and eight in South Ostrobothnia. In Päijät-Häme, ECEC centres were randomized (simple random allocation) into intervention and control arms using a computer-generated random sequence within four strata defined by the socio-economic profile of the areas provided by the municipality. In South Ostrobothnia, each municipality acted as an individual stratum. Among ECEC centres in this area, the randomization was not feasible due to practical reasons related to meal logistics and limited kitchen resources. Therefore, ECEC centres with an in-house kitchen were assigned to the intervention arm (n = 3). The ECEC centres without in-house kitchens (n = 5) served as controls, because food was transported to them. Blinding was not possible due to the nature of the intervention. Randomization was performed by the principal investigators in February 2022.

The overall aim of the study was to modify the food offerings in Finnish ECEC centres to better align with meal recommendations for ECEC [28] and to reduce the climate impact of ECEC food supply by providing children with healthy and environmentally more sustainable meals during a 10-month intervention period [27, 29]. The study was registered at ClinicalTrials (NCT05249946; https://clinicaltrials.gov). The intervention was conducted from March to December 2022. The effects of diet on bone and mineral metabolism markers and intakes of bone-related nutrients were of interest in this paper as secondary findings of the FoodStep trial. No power calculations were conducted.

### Ethical approval

This study was conducted according to the guidelines laid down in the Declaration of Helsinki, and all procedures involving human subjects were approved by the Ethics Committee of the Helsinki and Uusimaa Hospital District (1553/2021, 23 June 2021). Written informed consent was obtained from each child’s legal guardian for the child’s participation.

### Study population

**Fig. 1:**
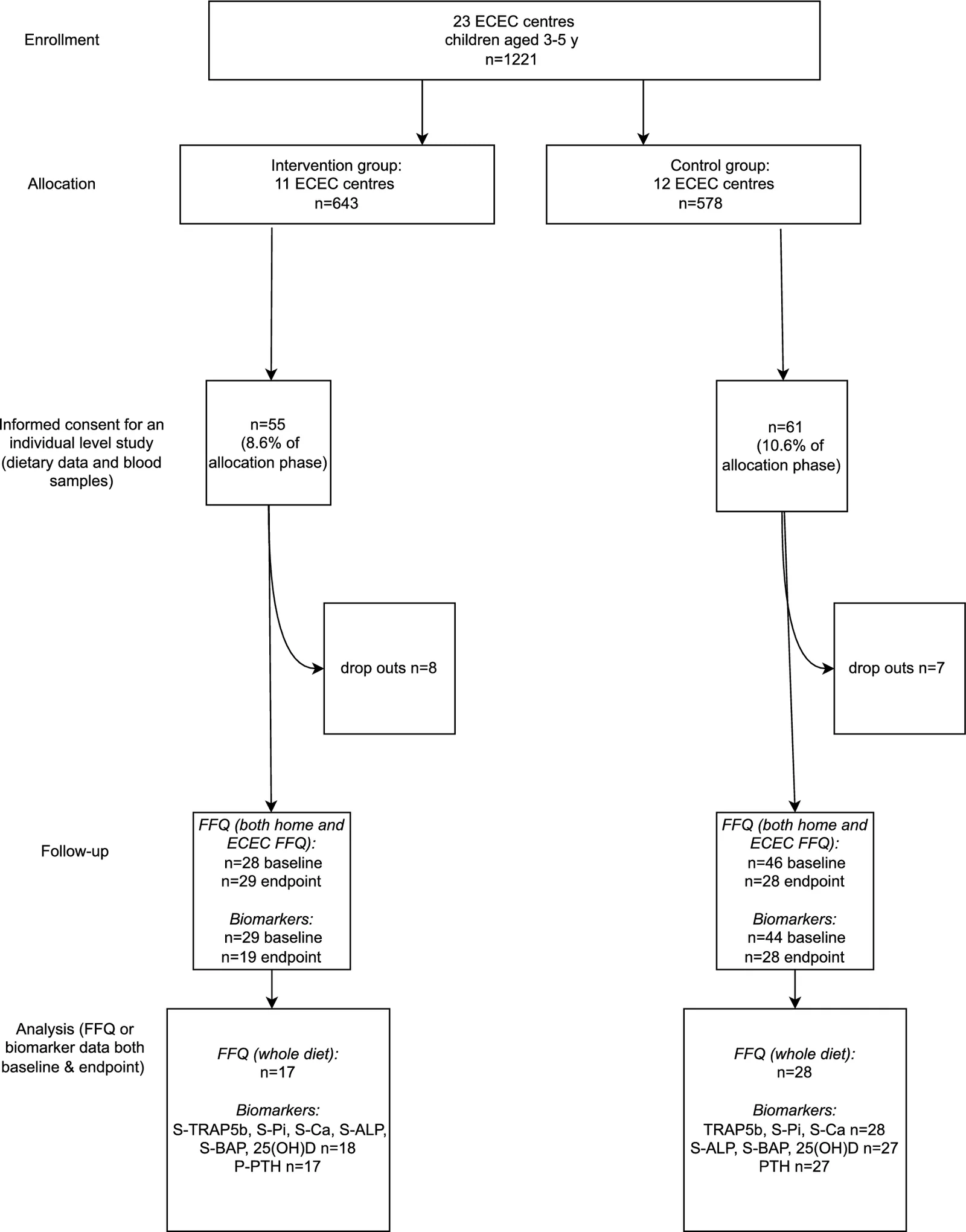
Enrolment of the FoodStep study. 25(OH)D 25-hydroxyvitamin D; *ECEC* Early childhood care and education; *FFQ* Food frequency questionnaire; P-*PTH* Plasma parathyroid hormone; *S-ALP* Serum total alkaline phosphatase; *S-BAP* Serum bone-specific alkaline phosphatase; *S-Ca* Serum calcium; *S-Pi* Serum phosphate; *S-TRAP5b* Serum tartrate-resistant acid phosphatase 5b

A total of 1221 children aged 3–5 years attended full-time daycare in autumn 2021 at 23 ECEC centres participating in the system-level intervention study during 2022. Of the children, 643 (53%) were in the intervention and 578 (47%) in the control ECEC centres (Fig. 1). All 3–5-year-old children in the participating ECEC centres were invited to a detailed study collecting individual-level information on diet and biomarkers. The ongoing COVID-19 protection measures prevented the research team from carrying out face-to-face recruitment in the ECEC centres. Therefore, legal guardians of the children were contacted through ECEC centres. Recruitment of the families took place between November 2021 and February 2022. Informed consent was obtained for 116 children (9.5% of those potentially invited): n = 55 in the intervention (8.6% of those potentially invited) and n = 61 (10.6% of those potentially invited) in the control ECEC centres. In this substudy related to bone health, we only used data of children whose blood samples or dietary data were available at both baseline and endpoint (see Fig. 1 for enrolment). Complete data were available for 32 subjects (intervention n = 13, control n = 19). Additionally, blood sample data only were available for 14 subjects (intervention n = 5, control n = 9), and dietary data only for 13 subjects (intervention n = 4, control n = 9). This resulted in total of 59 subjects (intervention n = 22, control n = 37) of whom 46 subjects had blood sample data (intervention n = 28, control n = 18) and 45 subjects had dietary data (intervention n = 17, control n = 28). Of the children with biomarker data, 63% originated from randomized ECEC centres, while the corresponding proportion for children with dietary data was 69%, respectively.

### Intervention components

The FoodStep intervention aimed to change the ECEC centre meal menus to align more closely with the National Meal Recommendations for ECEC [28] and to reduce the climate impact of the served and consumed meals [27]. The aim of the menu changes was to include increased servings of legumes, fruits, vegetables, and sustainable fish from nearby areas, while servings of red meat and processed meat products were decreased, along with moderated milk servings. Changes in menus are shown in detail elsewhere [29]. Supplemental Table 1 shows the principles of the menu modifications in the intervention ECEC centres. The ECEC intervention was co-created with professionals in the fields of ECEC and food services. The intervention menus were co-developed with food service professionals. Food education materials and training tailored to encourage acceptance of the menu changes were provided to the intervention ECEC centres [27, 30]. The control ECEC centres maintained their regular meals and usual food education.

### Background data

Background data were collected by electronic questionnaires filled in by guardians. Data on age, sex, and parental education level at study baseline are used in the current paper. The educational level was originally categorized into three groups: upper secondary education (no one announced having lower education), bachelor’s degree or similar, and master’s degree or higher. Due to the small number of subjects in the lowest category, it was combined with the middle category. The highest education in the family is reported here.

### Dietary assessment

Children’s diet was assessed at baseline, mid-term, and at the end of the intervention; here we use baseline and endpoint data. Electronic food frequency questionnaires (FFQs) were used to assess food consumption and nutrient intakes. Parents and ECEC personnel recorded children’s food intake on separate electronic FFQ forms assessing food intake at home (“home FFQ”) and at ECEC centres (“ECEC FFQ”), respectively. Parents and ECEC personnel received written instructions for filling in the FFQ.

The home FFQ for young children was developed by modifying a validated semiquantitative FFQ designed for women of reproductive age [31]. Food record data of 3–6-year-old Finnish children in two previous studies [32, 33] provided comprehensive information on the diets of Finnish children to support the development of the home FFQ. The home FFQ contained 150 rows (including different types of dietary supplements n = 8), for which the parents reported their child’s average food consumption outside ECEC hours during the past month. The ECEC FFQs were filled in by ECEC personnel and the instruction was to recall a child’s typical eating during the previous one to two months (as the menu cycles were from 5 to 6 weeks, depending on the ECEC centre). The ECEC FFQ was newly developed for this study. The municipalities’ food services provided information on ECEC menus, including breakfast, lunch, and afternoon snack, which were used to determine the serving frequencies of the main lunch dish types and side dish types appearing in the FFQ. The ECEC personnel reported portion sizes of 27 main dish types and eight side dish types for each child’s lunch meals. The FFQ contained also 79 other food items. To calculate food consumption and nutrient intake, recipes representing the average content of each queried food/drink/supplement were created based on a previous study of Finnish children’s diet [32] and ECEC menus. The data from the ECEC FFQ and home FFQ were combined for estimating total dietary intake. Details of the dietary assessment are presented in the Supplemental Material. In this paper, we report the daily intakes of macronutrients (E%), calcium (mg/MJ), vitamin D (µg/MJ), phosphorus (mg/MJ), and the molar dietary calcium-to-phosphorus ratio. Dietary fibre calculation was based on the internationally approved CODEX definition of fibre, and value 8 kJ/g was used to calculate fibre in E% [34]. In addition, total (per MJ) and supplemental (µg) vitamin D intakes are reported.

### Biomarker analyses

Blood samples collected in February–March 2022 and October–December 2022 were outsourced to local clinical laboratories. An overnight fast before blood sampling was advised. Serum and plasma were separated and frozen samples transferred to the Finnish Institution for Health and Welfare and stored at − 70 or − 80 °C until analysis. MicroVue enzyme-linked immunoassay kits (Quidel, San Diego, CA, USA) were used to analyse bone formation marker bone-specific alkaline phosphatase (S-BAP) and bone resorption marker tartrate-resistant acid phosphatase 5b (S-TRAP5b) concentrations in serum. Plasma intact PTH (P-PTH) was analysed by immunochemiluminometric method with a Siemens Atellica^®^ IM1600 analyser (Siemens, München, Germany). The S-25(OH)D concentrations were analysed using a chemiluminescent microparticle immunoassay (ARCHITECT 25-OH Vitamin D assay, Abbott Laboratories, Abbott Park, IL, USA) that measures S-25(OH)D_2_ and S-25(OH)D_3_. Serum Ca (S-Ca), phosphate (S-Pi), and total alkaline phosphatase (S-ALP) concentrations were analysed using a photometric method with an Indiko automatic analyser (Thermo Clinical Labsystems Oy, Espoo, Finland). Intra-assay CVs were ≤ 7.2% for S-BAP, ≤ 3.7% for TRAP5b, 4% for P-PTH, and ≤ 2.8% for S-Ca, S-Pi, and S-ALP. Inter-assay CVs were 2.7% for S-BAP, 11.3% for S-TRAP5b, 6% for P-PTH, and 2.9% for S-25(OH)D. For S-25(OH)D, bias compared with all-laboratory trimmed means in the Vitamin D International External Quality Assessment Scheme (DEQAS) was − 3 ± 4.5% (mean ± SD).

### Statistical analysis

Normality of the variables was assessed using the Kolmogorov–Smirnov test. To improve normality, log10-transformed variables were used for P-PTH, S-ALP, S-BAP, S-Pi, and the calcium-to-phosphorus ratio. All tests were considered significant at *p* < 0.05. Statistical analyses were performed with R Studio (version 4.4.3) and SPSS Statistics version 29 (IBM, New York, NY, USA).

To examine the effect of the intervention, we employed linear mixed models using biomarkers and bone-related nutrient intakes as outcomes. Sex (female, male), group (intervention, control arm), timepoint (baseline, endpoint), and interaction between group and time were included as fixed effects. Child ID was included in the models as the random effect. Models using stratum variable in addition to child ID as a random effect were also performed, but due to the small sample size model fits were unacceptable for some outcome variables because of inadequate variability. The inclusion or exclusion of stratum in the models did not affect the results, and thus, the final analyses were performed without the variable. We present unadjusted means and standard deviations of the outcomes and consider adjusted estimates and 95% confidence intervals (CIs) of the group*time interaction as representative of the effect of the intervention.

One subject from the control group was excluded from the S-BAP and S-ALP analyses due to pathologically high concentrations at the endpoint since this subject affected the normality of the variables even after log10 transformation. We also performed sensitivity analyses by excluding the subjects with non-fasting samples or samples taken after 10 am (intervention group n = 15–16, control group n = 22–23), but it did not affect the results, and thus, we decided to include these subjects in the analyses.

## Results

### Background characteristics

Baseline characteristics of the participating children stratified by the availability of biomarker or dietary data are described in Table 1. Sex distribution was approximately equal. Most of the children were 3 years old, and the majority of participants (~ 60%) were from the Päijät-Häme area. Parental education level was high since most of the parents had at least a bachelor’s level education.

**Table 1:** Background data of the FoodStep study participants at the baseline of the study

	Intervention group		Control group	
	Biomarker data (n = 18)	Dietary data (n = 17)	Biomarker data (n = 28)	Dietary data (n = 28)
*Sex (n/%)*				
Girl	8 (44.4%)	7 (41.2%)	15 (53.6%)	15 (53.6%)
Boy	10 (55.6%)	10 (58.8%)	13 (46.4%)	13 (46.4%)
*Age (n/%)*				
3 y	10 (55.6%)	10 (58.8%)	12 (42.9%)	8 (28.6%)
4 y	3 (16.7%)	4 (23.5%)	11 (39.3%)	11 (39.3%)
5 y	5 (27.8%)	3 (17.6%)	5 (17.9%)	9 (32.1%)
*Area (n/%)*				
Päijät-Häme	11 (61.1%)	9 (52.9%)	18 (64.3%)	22 (78.6%)
South Ostrobothnia	7 (38.9%)	8 (47.1%)	10 (35.7%)	6 (21.4%)
*Highest education in family (n/%)*				
Upper secondary school, bachelor's degree or equivalent	12 (66.7%)	14 (82.4%)	21 (75.0%)	21 (75.0%)
Master's degree or higher	6 (33.3%)	3 (17.6%)	7 (25.0%)	7 (25.0%)

### Bone turnover and mineral metabolism markers

**Fig. 2:**
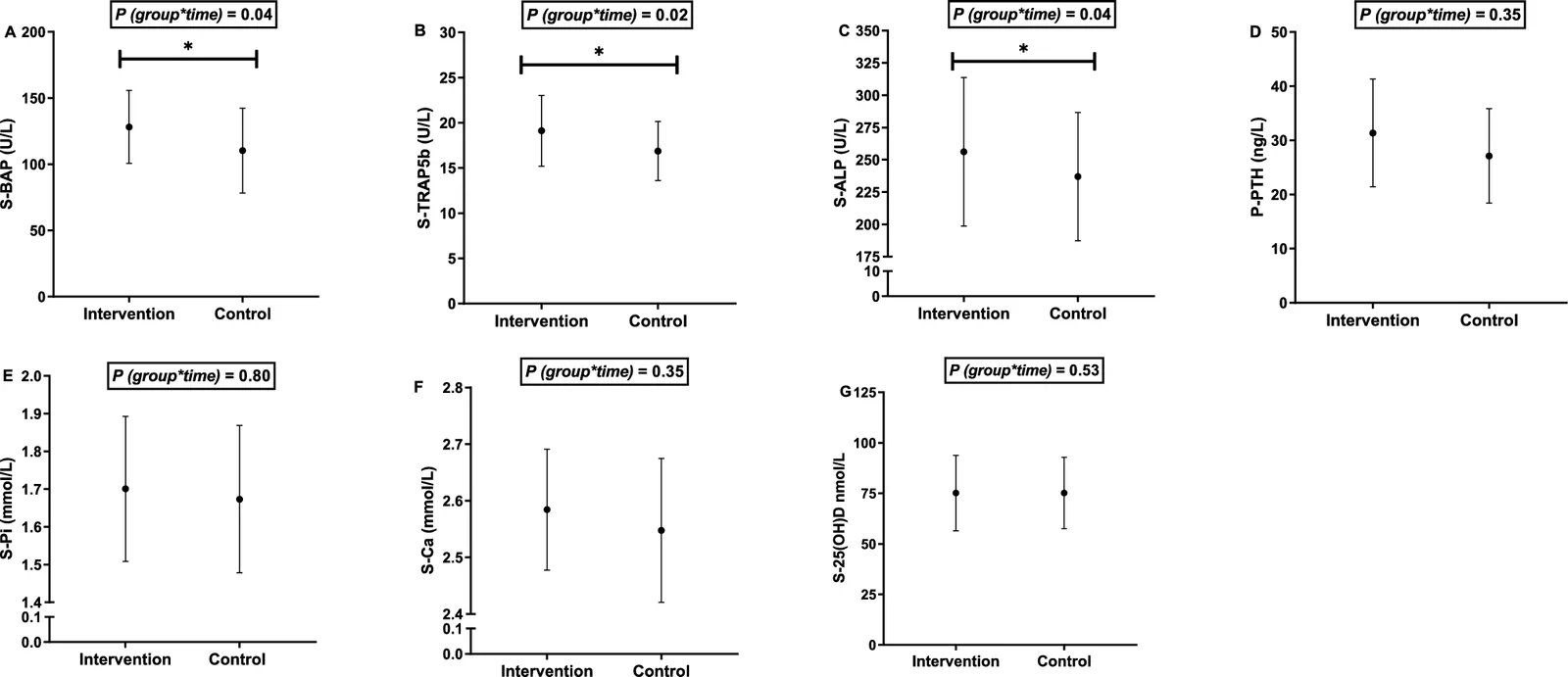
Circulating bone and mineral metabolism marker concentrations (mean with standard deviation) among children of the FoodStep study in the intervention (n = 18) and control (n = 28) groups at the endpoint of the 10-month intervention. **A** Serum bone-specific alkaline phosphatase (S-BAP), **B** Serum tartrate-resistant acid phosphatase 5b (S-TRAP5b), **C** Serum total alkaline phosphatase (S-ALP), **D** Plasma parathyroid hormone (P-PTH), **E** Serum phosphate (S-Pi), **F** serum calcium (S-Ca), **G** Serum 25-hydroxyvitamin D (S-25(OH)D). *p* values for interaction (group*time) from mixed model for analysis (sex, intervention group, timepoint, and interaction group*time as fixed effects; child ID as random effect). For PTH, n = 17 in the intervention group. For S-ALP, S-BAP, S-25(OH)D, n = 27 in the control group

Concentrations of bone resorption marker S-TRAP5b (estimate 2.12 U/L, 95% CI 0.35; 3.90, group*time *p* = 0.02) and bone formation marker S-BAP (log-transformed estimate 0.05 U/L, 95% CI 0.004; 0.104, group*time *p* = 0.04) as well as total S-ALP (log-transformed estimate 0.034 U/L, 95% CI 0.002; 0.066, group*time *p* = 0.04) were higher in the intervention group at study endpoint (Fig. 2A–C). The intervention showed no effect on the concentrations of P-PTH, S-Pi, S-Ca, or S-25(OH)D (Fig. 2D–G). Supplemental Table 2 provides more detailed information.

None of the children had vitamin D deficiency (S-25(OH)D < 30 nmol/L) [4], but at baseline two children (11.1%) in the intervention group, and one child (3.8%) in the control group had inadequate vitamin D status (S-25(OH)D 30–49.9 nmol/L) [4], the frequencies being one (5.6%) and one (3.8%) at the endpoint, respectively.

### Bone-related nutrient intakes

We found no intervention effect on the intakes of energy or macronutrients (E%) or the intakes of bone-related nutrients (vitamin D, calcium, or phosphorus in relation to energy intake, or molar calcium-to-phosphorus ratio) at the study endpoint (Table 2). Vitamin D supplement use was common; all children, except for one child in the control arm at baseline and another child at the endpoint, used supplements (data not shown).

**Table 2:** Unadjusted mean (SD) daily energy and nutrient intakes of the participants at the baseline and at the end of the intervention of the FoodStep study (n = 45) and results for the mixed model analysis

	Unadjusted results								Mixed model results				
	Intervention group (n = 17)				Control group (n = 28)								
	Baseline		Endpoint		Baseline		Endpoint		Estimate	SE	Lower 95% CI	Upper 95% CI	P (group*time)
	Mean	SD	Mean	SD	Mean	SD	Mean	SD					
Energy (kJ/d)	7300	1831	7158	2044	8380	1501	7679	1947	560	489	-399	1518	0.26
Protein (E%)	15.8	1.62	15.2	1.71	15.2	1.46	15.1	1.47	–0.06	039	-1.38	0.14	0.12
Carbohydrates (E%)	49.8	3.06	50.8	4.00	51.8	3.31	51.7	2.61	1.08	0.87	-0.62	2.79	0.22
Fat (E%)	32.1	3.89	31.7	4.41	30.5	3.14	30.7	2.35	–0.67	1.00	-2.63	1.30	0.51
Fibre (E%)	2.26	0.32	2.36	0.31	2.57	0.48	2.47	0.33	0.20	0.11	-0.02	0.42	0.08
Vitamin D													
*Dietary (μg/MJ/d)*	1.83	0.40	1.64	0.36	1.75	0.42	1.60	0.42	–0.05	0.11	-0.27	0.18	0.70
*Total (diet* + *supplement) (μg/MJ/d)*	3.31	0.65	3.46	0.87	2.92	0.93	2.83	0.89	0.02	0.03	-0.03	0.07	0.36
*Supplemental (μg/d)*	10.5	3.84	11.4	5.20	9.91	4.73	8.63	4.11	2.15	1.48	-0.76	5.05	0.16
Calcium, dietary (mg/MJ/d)	183.1	49.4	160.5	42.8	165	30.6	161	38.8	–19.1	12.5	-43.6	5.48	0.14
Phosphorus (mg/MJ/d)	198.6	31.9	189.5	30.6	194	22.6	188	21.3	–2.77	7.23	-16.9	11.4	0.70
Calcium-to-phosphorus ratio (mol/mol)*	0.73	0.13	0.69	0.14	0.67	0.11	0.67	0.15	–0.03	0.02	-0.08	0.01	0.18

*Calcium-to-phosphorus ratio *p* value and estimate from log10-transformed analysis

Mixel model for analysis: Sex, group, timepoint, and interaction group*time as fixed effects; child ID as random effect

### Discussion

The cluster-randomized 10-month intervention study investigating the switch towards more environmentally sustainable, plant-forward diets in the Finnish ECEC centres showed a significant effect on bone turnover markers of initially 3–5-year-old children. The intervention arm had higher bone formation and resorption marker concentrations, indicating accelerated bone turnover despite adequate mean vitamin D status (25(OH)D > 50 nmol/L) and no changes in the intakes of key bone-related nutrients: calcium, vitamin D, and protein. No effects on vitamin D status or circulating PTH, calcium, or phosphate concentrations were observed.

To the best of our knowledge, this is the first time that the effects of a switch towards a more sustainable diet on bone turnover in an ECEC setting were studied. Our results showing higher bone formation marker S-BAP, total S-ALP and resorption marker S-TRAP5b concentrations throughout the intervention were mostly in line with previous results on vegetarian diets in 7–10-year-old children conducted in Poland [18, 20, 21, 35]. Higher concentrations of bone resorption markers [18, 20, 21] as well as PTH [35] have been observed among vegetarians than among omnivores, while findings for bone formation markers have been inconsistent [18, 20, 21]. However, a major difference between the Finnish and Polish context is the vitamin D food fortification practices and generally higher vitamin D intakes as a consequence of vitamin D supplementation recommendations in Finland [23], that were well adapted also in the current study population. In a recent study on 2–7-year-old Finnish children following vegan, vegetarian, and omnivorous diets with, on average, adequate vitamin D and calcium intakes, a linear trend towards higher PTH concentrations with more plant-based diets was observed, whereas no differences between diet groups in either bone formation or bone resorption markers were present [22]. This raises the question of other underlying factors for bone health besides the traditional vitamin D and calcium intakes.

Higher bone turnover marker concentrations were seen here in the intervention arm without observing any differences in key bone nutrient intakes (calcium, vitamin D, protein) or in vitamin D status, while in the earlier, above-mentioned study [22] protein intake (E%) was lower in the vegan and vegetarian groups than in the omnivorous group. It is possible that the increased share of plant-sourced proteins consumed in the intervention arm may play a role in the present results; plant-based proteins lag behind animal-based ones in quality due to lower digestibility and more imbalanced amino acid composition in relation to physiological needs [36]. The role of plant and animal-based proteins on bone health has been discussed, but no consensus has been reached; most randomized controlled trials regarding plant proteins and bone health have used soy protein [37]. This impairs the generalizability of the results to other plant protein sources because the quality of soy protein is comparable to animal-based proteins, unlike many other plant proteins [37]. At a mechanistic level, dietary protein promotes bone mineralization by enhancing intestinal calcium absorption and increasing circulating insulin-like growth factor 1 (IGF-1), which subsequently stimulates osteoblastic activity and bone formation [38]. IGF-1 is an important factor in longitudinal bone growth and bone mass acquisition during childhood [39]. Analysis of this growth factor as well as information on the share of animal and plant-based proteins would have provided more insights into bone status and the role of protein quality since higher animal protein consumption has been associated with IGF-I concentrations [40–42].

Our study did not show an effect of transition to more plant-based ECEC centre meals on the intakes of vitamin D and Ca intakes, the key bone nutrients. This is in contrast to the finding of a recent systematic review comparing plant-based and omnivorous diets, which concluded that children and adolescents were at risk of inadequate vitamin D and Ca intakes, regardless of their diet [43]. The difference is probably due to Finland’s wide national vitamin D food fortification policy of fluid milks, their plant-based alternatives, and fat spreads [44]. Moreover, there are supplementation recommendations, in particular for vulnerable groups; vitamin D supplements are recommended for all individuals < 18 years of age [23]. Vitamin D policies have improved vitamin D intakes and status in Finland [45, 46]. However, moderating the consumption of dairy products can be challenging since they play a central role in the current Finnish diet and food culture [47] They also serve as a key source of calcium and, when fortified, of vitamin D. Fortunately, most plant-based drinks are nowadays fortified with calcium and vitamin D in Finland [22], making them suitable milk alternatives from the bone nutrient perspective. Calcium, phosphorus, and vitamin D intakes (including also supplements) in our study were close to the values previously observed among 3–6-year-old Finnish children, which were calculated based on 4-day food records [48], although we used FFQ. Also, the molar calcium-to-phosphorus ratio was close to the ratios observed in the previous study comparing plant-based and omnivorous diets in children [22]. This probably accounts for our finding of not observing any effects of the intervention on serum calcium or phosphate concentrations.

It is possible that the changes in bone turnover marker concentrations in the whole study population are partly explained by growth during the 10-month intervention period. Our study participants were in their early childhood when both bone modelling, i.e. formation and shaping of bone, and bone remodelling, i.e. replacement of old bone with new bone, occur, the predominant stage of bone turnover being bone modelling [49]. This is a critical period during which bone grows in both shape and size, establishing a basis for adequate bone mass accumulation. Among children, bone turnover markers correlate with height velocity, thus they may reflect the cumulative effect of bone growth and remodelling [50]. The lack of population-based bone turnover marker reference values for children has been recognized [50], which also confounds the conclusions of the current study: what is the clinical relevance? Recently, an international position paper suggested broadening the use of bone turnover markers for adults and acknowledged the need for standardized assays of S-TRAP5b and S-BAP in monitoring specific osteoporosis conditions [51]. Thus, more standardized methods are expected in the future, along with studies that provide reference values for paediatric populations.

In Finland, the ECEC services include daily meals (such as breakfast, lunch, and an afternoon snack) as part of the overall provision, with no additional charge beyond the standard client fee determined by family income and size [24]. The National Meal Recommendations for ECEC are recommended in the planning of municipal food services [28]. The feasibility of sustainable dietary changes in ECEC centres depends on the availability of nutritionally equivalent alternatives at comparable costs and children's acceptance of these foods. In Finland, fortified plant-based dairy alternatives offer suitable milk replacements, while meat can be substituted with fish or legumes. However, challenges like cost and children’s food preferences require further attention. The current intervention included both dietary modifications and educational components, which together likely contributed to the observed effects. However, in the intervention ECEC centres, the food education component was only partially implemented [30]. In contrast, the provision of main meat dishes decreased, while that of vegetarian dishes increased across all intervention centres [29]. The ECEC meal criteria currently being revised in Finland offer great potential for sustainability integration, as all children in public ECEC are served food prepared according to the same meal recommendations and do not bring their own packed lunches. Obviously, adequate intakes of certain nutrients, such as vitamin D, need to be ensured through home-based supplementation practices; this was implemented well for the participants of this study. In countries with a lower level of governmental fortification regulations, this kind of intervention requires additional input to ensure the nutritional adequacy of the ECEC meals.

To our knowledge, this is the first intervention study investigating a shift towards more plant-based meals and the effects on bone turnover in children; previous studies on plant-based diets have used cross-sectional datasets. The strength of this study was the ECEC centre-based approach, which seemed to be feasible in introducing more environmentally sustainable food sources and menus to young children [29]. Two separate FFQs that considered the menu served at each participating ECEC centre and the time that a child spent in daycare provided more detailed information on children’s diets than one simple FFQ. Thus, our approach allowed comprehensive data collection on the child’s total diet as opposed to one FFQ relying on information provided only by the parents. However, this study is not without limitations, the main one being the recruitment period during the COVID-19 pandemic, which limited research personnel visits to the ECEC centres. This probably somewhat decreased the number of participants in the individual-level study that included blood sampling and dietary data collection. The small sample size combined with the FFQ method, which do not allow detection of subtle differences between individuals, may limit the power of the analyses. The effects of intervention on bone turnover markers were somehow marginal (*p* ≥ 0.02), and it is also possible that the detected differences in bone turnover markers may not be of long-term clinical relevance since the children were at the stage of rapid growth characterized also by rapid bone metabolism. It is possible that the ECEC centres in the intervention arm differed from those in the control arm as randomization was only feasible in one of the four municipalities. Nevertheless, most participants (69% with dietary data and 63% biomarker data) were from the municipality where the randomization procedure was implemented, reducing the likelihood of systematic bias. The small sample size affected the fit of the statistical models by limiting within-group variation, necessitating omission of the stratum (“cluster”) variable from the models. This may have led to underestimated standard errors and potentially inflated statistical significance, however, the results remained unchanged when the variable was excluded. Moreover, the FFQ result was a combination of two FFQs, one filled in at the ECEC centre and the other at home, and it was not validated against food records among young children. Lastly, a drawback of the study was the use of bone turnover markers over bone mineral density assessment, limiting conclusions that could be drawn about longer-lasting effects of plant-forward daycare meals on bone health.

## Conclusions

The results of this 10-month cluster-randomized intervention trial suggest accelerated bone turnover in terms of higher bone resorption and bone formation among young children when transitioning to plant-forward meals at ECEC centres. This phenomenon was observed despite finding no effects on vitamin D status or intakes of the key bone-related nutrients of calcium, vitamin D, and protein. However, the clinical relevance of the results provided by this small dataset remains unclear. Given that the results may suggest potential harmful effects on long-term bone health caused by a shift towards more plant-based diets, further investigation of such underlying factors as protein quality is warranted.

## Supplementary Information

Below is the link to the electronic supplementary material.

Supplementary Table 1 (DOCX 16 KB)

Supplementary Table 2 (DOCX 20 KB)

Supplementary Material Dietary Assessment (DOCX 20 KB)

## Data Availability

Researchers interested in the data from this study may contact principal investigator Suvi M. Virtanen, suvi.virtanen@tau.fi.

## Abbreviations

25(OH)D: 25-hydroxyvitamin D
Ca: Calcium
Ca:P ratio: Calcium-to-phosphorus ratio
ECEC: Early childhood education and care
FFQ: Food frequency questionnaire
P: Phosphorus
PTH: Parathyroid hormone
P-PTH: Plasma parathyroid hormone
S-25(OH)D: Serum 25-hydroxyvitamin D
S-ALP: Serum total alkaline phosphatase
S-BAP: Serum bone-specific alkaline phosphatase
S-Ca: Serum calcium
S-Pi: Serum phosphate
S-TRAP5b: Serum tartrate-resistant acid phosphatase 5b
