# Supplementary material for: A 10-month cluster-randomized trial to shift towards plant-forward meals in early childhood education and care centres: effects on bone and mineral metabolism in Finnish children
Source: Eur J Nutr. 2026 Jul 23;65(5):219. doi: 10.1007/s00394-026-04063-y (PMC13395845; doi:10.1007/s00394-026-04063-y)
Supplement: Supplementary file 1 — Supplementary Table 1 (DOCX 16 KB) [file 394_2026_4063_MOESM1_ESM.docx]

**Supplemental Table 1. The aims of the FoodStep intervention menu changes at early care and education centres.**

| ***Dietary component*** | ***Aim*** | ***Examples*** |
| --- | --- | --- |
| Red and processed meat | Reduced use of red meat | Red meat up to 1 times per week  No processed meat |
| Dairy | Rationalized use of milk | No milk as a drink when another dairy product is served on a meal  Water, not fluid milk, used as a thirst quencher |
| Vegetables, fruits and berries | Increasing and diversified use of vegetables, fruits, and berries | A child’s fist-size portion in each meal  Options based on seasonal availability |
| Fish | Increased use of sustainable fish species | WWF fish guide, green-rated fish species  Fish main dish 1–2 times/wk |
| Legumes | Increased use of legumes | 4 times/week |
