# Supplementary material for: A 10-month cluster-randomized trial to shift towards plant-forward meals in early childhood education and care centres: effects on bone and mineral metabolism in Finnish children
Source: Eur J Nutr. 2026 Jul 23;65(5):219. doi: 10.1007/s00394-026-04063-y (PMC13395845; doi:10.1007/s00394-026-04063-y)
Supplement: Supplementary file 2 — Supplementary Table 2 (DOCX 20 KB) [file 394_2026_4063_MOESM2_ESM.docx]

| Supplemental Table 2. Unadjusted mean (SD) blood biomarker concentrations of the participants at the baseline and the end of the intervention of the FoodStep study (n=46) and results for the for mixed model analysis. | | | | | | | | | | | | | | |
| --- | --- | --- | --- | --- | --- | --- | --- | --- | --- | --- | --- | --- | --- | --- |
|  | **Unadjusted results** | | |  |  | | |  |  | **Mixed model results** | | | | |
|  | *Intervention group (n=18*)* | | |  | *Control group (n=28†)* | | |  |  |  |  |  |  |  |
|  | Baseline |  | Endpoint | | Baseline |  | Endpoint |  |  |  |  |  |  |  |
|  | Mean | SD | Mean | SD | Mean | SD | Mean | SD |  | Estimate | SE | Lower 95% CI | Upper 95% CI | P (group*time) |
| Serum bone-specific alkaline phosphatase (U/L) | 134.7 | 38.6 | 126.7 | 27.5 | 130.5 | 34.5 | 110.3 | 32.0 |  | 0.05 | 0.03 | 0.00 | 0.10 | 0.04 |
| Serum tartrate-specific acid phosphatase 5B (U/L) | 20.7 | 3.03 | 19.1 | 3.91 | 20.6 | 4.14 | 16.9 | 3.25 |  | 2.13 | 0.91 | 0.35 | 3.90 | 0.02 |
| Serum total alkaline phosphatase (U/L) | 249.4 | 66.3 | 256.2 | 57.5 | 248.6 | 52.8 | 237.0 | 49.7 |  | 18.4 | 8.91 | 0.91 | 35.8 | 0.04 |
| Plasma parathyroid hormone (ng/L) | 31.1 | 12.0 | 31.4 | 9.94 | 29.5 | 10.4 | 27.1 | 8.73 |  | 2.70 | 3.07 | -3.32 | 8.73 | 0.30 |
| Serum 25-hydroxyvitamin D (nmol/L) | 82.2 | 39.4 | 82.2 | 34.7 | 76.4 | 19.5 | 76.0 | 17.8 |  | 0.43 | 6.32 | -12.0 | 12.8 | 0.95 |
| Serum phosphate (mmol/L) | 1.66 | 0.13 | 1.70 | 0.19 | 1.62 | 0.12 | 1.67 | 0.20 |  | -0.02 | 0.06 | -0.14 | 0.11 | 0.80 |
| Serum calcium (mmol/L) | 2.53 | 0.07 | 2.55 | 0.06 | 2.52 | 0.07 | 2.50 | 0.08 |  | 0.04 | 0.04 | -0.04 | 0.13 | 0.35 |
| Total and bone-specific alkaline phosphatase, parathyroid hormone, and phosphate P values from log10-transformed analysis; bone-specific alkaline phosphatase estimate from log values | | | | | | | | | | | | | |  |
| Mixel model for analysis: Sex, group, timepoint, and interaction group*time as fixed effects; child ID as random effect | | | | | | | | | | | | | |  |
| Bone-specific alkaline phosphatase *n=18 †n=27; parathyroid hormone *n=17 †n=27; 25-hydroxyvitamin D *n=18 †n=27 | | | | | | | | | | | | | |  |
|  |  |  |  |  |  |  |  |  |  |  |  |  |  |  |
